# Supplementary material for: Arabidopsis thaliana G3BP Ortholog Rescues Mammalian Stress Granule Phenotype across Kingdoms
Source: Int J Mol Sci. 2021 Jun 11;22(12):6287. doi: 10.3390/ijms22126287 (PMC8230867; doi:10.3390/ijms22126287)
Supplement: Supplementary file 1 [file ijms-22-06287-s001.zip › ijms-1205687-supplementary.pdf]

```
AtG3BP-1     1 ----MAQQEASPSPGAEVVGRAFVEQYYHILHQSPGLVHRFYQDSSFLTRPDVTGA---V
AtG3BP-2     1 --------MATPYPGATQVGSYFVGQYYQVLQQQPDLIHQFYSEPSRAIRIDGDST----
AtG3BP-3     1 MAMLGAQQVPAAACTPDMVGNAFVPQYYHILHQSPEHVHRFYQEISKLGRPEENGL---M
AtG3BP-4     1 ------MATEGVVPSAQDIAAEFVRQYYHVLGQLPHEARRLYVDASVVSRPDVTGT---M
AtG3BP-5     1 ----MALESNAPVVDPNTIGNSFVEKYYNLLYKSPSQVHQFYLDDSVLGRPGSDGE---M
AtG3BP-6     1 ----MTPESNAPSVDPQFVGNGFVQEYYNHLYDSTSEVHKFYLEDSMISRPGLDGE---I
AtG3BP-7     1 ---MDSTAATKRVVDPLTVGSAFVNQYYYIFCNMPEHLPRFYQEISRVGRVGQDGV---M
HsG3BP-1     1 --------MVMEKPSPLLVGREFVRQYYTLLNQAPDMLHRFYGKNSSYVHGGLDSNGKPA
HsG3BP-2a    1 --------MVMEKPSPLLVGREFVRQYYTLLNKAPEYLHRFYGRNSSYVHGGVDASGKPQ
HsG3BP-2b    1 --------MVMEKPSPLLVGREFVRQYYTLLNKAPEYLHRFYGRNSSYVHGGVDASGKPQ
consensus    1         .    ... ...   ** .**  .. . .. ....* . * . ......    .

AtG3BP-1    54 TTVTTMQAINDKILSLKYE-DYTAEIETADAQESHERGVIVLVTGRLTGNDNV-RKKFSQ
AtG3BP-2    49 ETANSILHIHNMVMSLNFT---AIEVKTINSVESWEGGVLVVVSGSVKTKEFSNRRSFVQ
AtG3BP-3    58 SITSTLQAIDKKIMALGYG-VISAEIATVDTQESHGGGYIVLVTGYLTGKDSV-RRTFSQ
AtG3BP-4    52 MSFTSVEAINKHILSCDFE-NTKFEVLSVDSQNSLEDGIFIMVIGFMTGKDNQ-RRKFSQ
AtG3BP-5    54 VSVKSLKAINEQIMSFDYE-ISKIQILTADSQASYMNGVVTLVTGLLTVKEGQ-RMRFSQ
AtG3BP-6    54 VTIKSLKGINDQIMSIDYK-SSRIEILTADSQSTLKNGVVTLVTGLVIGNDGG-RRKFSQ
AtG3BP-7    55 RDFSTFQGISEELKRLTYGDCNSAEITSYDTQESHNGGFLLFVTGYFTLNERS-RRKFTQ
HsG3BP-1    53 DAVYGQKEIHRKVMSQNFT-NCHTKIRHVDAHATLNDGVVVQVMGLLSNNNQA-LRRFMQ
HsG3BP-2a   53 EAVYGQNDIHHKVLSLNFS-ECHTKIRHVDAHATLSDGVVVQVMGLLSNSGQP-ERKFMQ
HsG3BP-2b   53 EAVYGQNDIHHKVLSLNFS-ECHTKIRHVDAHATLSDGVVVQVMGLLSNSGQP-ERKFMQ
consensus   61  .. ....* ...... .    . .. ..... ..  *....*.*...  ..  ...*.*

AtG3BP-1   112 SFFLAPQDK---GYFVLNDVFRFLEE------KEVTAQARSVPINGTTRDVQAPIEPER-
AtG3BP-2   106 TFFLAPQEK---GYFVLSDVFLFVDEG-----TVYYHQPSYLSEIKHEAQLNPPTRHPDP
AtG3BP-3   116 TFFLAPQET---GYFVLNDMFRFIDEGTVVHGNQIPVNNVQAPVNTYQDTAAAKEIPDDF
AtG3BP-4   110 MFYLARQN----TLVVLNDMLRYVD---------QEDSSTTETPCEPVTEIVRPADGLK-
AtG3BP-5   112 SFFLVPLNG---SYFVLNDVFRYVAD------EIVEPEANKKEVEEVIPQVVQPTEQVD-
AtG3BP-6   112 SFFLVSRNG---SYFVLNDTFRYVSD------EFVEPEAT-KEVEESQ-STNAITEPAN-
AtG3BP-7   114 TFFLAPQEK---GFFVLNDILRFVNDDAKDNVPETIDGEVVSGINSTTPTIINGMKGSE-
HsG3BP-1   111 TFVLAPEGSVANKFYVHNDIFRYQDEVFGGFVTEPQEESEEEVEE-PEERQQTPEVVPDD
HsG3BP-2a  111 TFVLAPEGSVPNKFYVHNDMFRYEDEVFGDSEPELDEESEDEVEEEQEERQPSPEPVQEN
HsG3BP-2b  111 TFVLAPEGSVPNKFYVHNDMFRYEDEVFGDSEPELDEESEDEVEEEQEERQPSPEPVQEN
consensus  121 .*.*...      ..*..*.......      .....    ...    .  .    .

AtG3BP-1   162 ---VVVSHEPEVEPEPVASIEEEDLDNVAEVYD--PSDKDEGVVVDVEPIEPP-------
AtG3BP-2   158 QVSDYVLEEEASDYVNAVQIKDDLVDKYSLQEDQHQPQHEDYEDEVAIEETPREEVAVDV
AtG3BP-3   173 VQEKYVQENHAVKQTEVLSKSINEPEKVFTPSEDEQVSAAEEALVTETVNEAP-------
AtG3BP-4   156 -----KAEKTELKQKNVASVEKSVNAAVEKNAA---PLDNGKMKQSEKAVITQ-------
AtG3BP-5   162 -----EVAEPVTIPTQQPEAKQTTENTVKKPER---AVANGHPKTQEDNVVND-------
AtG3BP-6   160 -----ESVEAVIVPT---EAKTT----VTKPAS---AIPNGHAKVPEEKVVNE-------
AtG3BP-7   170 -QAACVSVNPVCKEVSKPLDNENAKDNVLVP-----EIANEVARTEITCKEVA-------
HsG3BP-1   170 SGT-FYDQAVVSNDMEEHLEEPVAEPEPDPEPEPEQEPVSEIQEEKPEPVLEETAPEDAQ
HsG3BP-2a  171 ANSGYYEAHPVTNGIEEPLEESSHEPEPEPESETKTEELKPQVEEKNLEELEE-------
HsG3BP-2b  171 ANSGYYEAHPVTNGIEEPLEESSHEPEPEPESETKTEELKPQVEEKNLEELEE-------
consensus  181      .    ...  .     . .    .     .       .          .. .

AtG3BP-1   210 TQISHNEILSVPQGDAPKHSYASILKQMKSSP--------------------APTTHVAR
AtG3BP-2   218 VHEHRAAPVEEPVGEKSKMSYASILKVAKEAATVP---------------VAATQPSYNK
AtG3BP-3   226 IEVQKVGESDSRTGEIPKRSYASIVKVMKEN---------------------AAPMSASR
AtG3BP-4   201 KVTEPDAAP---QPDGAKRSFADIVGSMAKN---------------------AAPFQVKS
AtG3BP-5   207 KSNG---------VDAPKKSFAHIVQDLAQN---------------------GATFNAKA
AtG3BP-6   198 NSSLPKAAEAKLQEEVPKKSFALIVQSLAQS---------------------AGTLQVKA
AtG3BP-7   217 DDSQKNYDPDDGLADAPKKSYASVLKVTKDK---------------------FGVPAVSL
HsG3BP-1   229 KSSSPAPADIAQTVQEDLRTFSWASVTSKNLPPSGAVPVTGIPPHVVKVPASQPRPESKP
HsG3BP-2a  224 KSTTPPPAEPVSLPQEPPKAFSWASVTSKNLPPSGTVSSSGIP-SHVKAPVSQPRVEAKP
HsG3BP-2b  224 KSTTPPPAEPVSLPQEPP----------------------------------KPRVEAKP
consensus  241 ... .  .      . ...... ..  ..                       ..    .
```

```
AtG3BP-1  250 NKPRPAPVNQKLTAP-------------------PAEPAARPEASAHENVPNSSHVDVE-
AtG3BP-2  263 SSQDINEWDQPMRTPSPQ----------LAAPLAPIQQSNSSTYVSDYGAEAEDGSGFED
AtG3BP-3  265 TPTKVEPKKQEDQAIHIP---------------LPTPLSEKSDSGANVAVNENNQ-ENER
AtG3BP-4  237 PVQAPVQKPKYVGQP--------------------RAAAAPQKPAYVSKSIKKNDQKVIE
AtG3BP-5  237 SPAKPKSKP--VTKP--------------------SAARESKAPAPVSEHSS-AATIDQQ
AtG3BP-6  237 SPVK--RKP--VEKP--------------------VAAPERKAPSPIRKQAS-AESIKPQ
AtG3BP-7  256 PSPKKIPKDQEHQAPSDPSTGQILKDQGQQASSDPSQVIESDTVSESVDASENGHNQEAV
HsG3BP-1  289 ESQIPPQRPQRDQRVREQ------------RINIPPQRGPRPIREAGEQGDIEPRRMVRH
HsG3BP-2a 283 EVQSQPPR-VREQRPRE-------------RPGFPP-RGPRPGRGDMEQNDSDNRRIIRY
HsG3BP-2b 250 EVQSQPPR-VREQRPRE-------------RPGFPP-RGPRPGRGDMEQNDSDNRRIIRY
consensus 301   ..  .. . ....                   .   . .   . .       . ..

AtG3BP-1  290 DDGHSIYVRNLPFDSTPTQLEEVFKNFGAIKHEGIQVR--SNKQQGFCFGFVEFETSSGK
AtG3BP-2  313 FEFKSVYVRNLPSDISASEIEEEFKNFGTIKPDGVFLR-TRKDVMGVCYAFVEFEDMTSV
AtG3BP-3  309 ALGPSIYLKGLPLDATPALLENEFQKFGLIRTNGIQVR--SQK--GFCFGFVEFESASSM
AtG3BP-4  277 VPGTSIFVANLPLNAMPPQLFELFKDFGPIKENGIQVRSSRGNANPVCFGFISFETVASV
AtG3BP-5  274 AEGYTIFVANLLMDATPEQLNETFKGFGAITKDGIQVRSYRLKGN--CFGFVTFASAEAV
AtG3BP-6  272 AQGSSIFVANLPMDATIEQLYETFKSFGAIRKDGIQVRSYPEKKN--CIGFVAFENGEAV
AtG3BP-7  316 AEGTSIYVRHLPFNANIDMLEAEFKQFGAITNGGIQVI--NQRGLGYPYGFVEFEEADAA
HsG3BP-1  337 PDSHQLFIGNLPHEVDKSELKDFFQSYGNVVELRINS---GGKLPN--FGFVVFDDSEPV
HsG3BP-2a 328 PDSHQLFVGNLPHDIDENELKEFFMSFGNVVELRINTKGVGGKLPN--FGFVVFDDSEPV
HsG3BP-2b 295 PDSHQLFVGNLPHDIDENELKEFFMSFGNVVELRINTKGVGGKLPN--FGFVVFDDSEPV
consensus 361  .........*. ...   . . *. .* ..  .....    .    ...*. *.. . .

AtG3BP-1  348 QSALEAS---PVTIGDRQAVVEEKKT--NSRGGGNNGGSRGRYFSGRGSFRNESFKGGRG
AtG3BP-2  372 ENAIKAS---PIYLGGRQVYIEERRP--NPAGVRGARRGGG----RGRGGYPTEAPRGRF
AtG3BP-3  365 QSAIEAS---PVMLNGHKVVVEEKRS--TARGNYRGRSTFG---VNTGYRNEGGRGRGSF
AtG3BP-4  337 QSVLQAAKNTPFMLADRKLRVKEKEV--DYDGSKPSGKTKGGSNKTQNGSADSSKTENGS
AtG3BP-5  332 KLVLQAHKESAIRIGNRRVSIEEKRG-NNDNG-RPAMRN-GGYRNDNGYRNDGYRPRGNG
AtG3BP-6  330 KNVFQAHRESPIRIGNRRASIEEKRGGNNQNGNRVSTRNNSGYKNEDGFRRDGYKPRGSG
AtG3BP-7  374 HRAIEAS---PVKIGGLRAFVEEKLS--TSRG-KRGNGNVG---YGNRNVGVGMRGRGSY
HsG3BP-1  392 QKVLSNR--PIMFRGEVRLNVEEKKT--RAARE-------GDRRDNR--LRGPGGPRGGL
HsG3BP-2a 386 QRILIAK--PIMFRGEVRLNVEEKKT--RAARERETRGGGDDRRDIRRNDRGPGGPRGIV
HsG3BP-2b 353 QRILIAK--PIMFRGEVRLNVEEKKT--RAARERETRGGGDDRRDIRRNDRGPGGPRGIV
consensus 421 . .. ..   .. ...... ..*...     . .      .  .      .   ....

AtG3BP-1  403 GGGRGGYGRGGGEFSGRPKSSN-------PRNGGEGYQRVPQNGGGGRGGRGEGGRGGAR
AtG3BP-2  423 GGRGSGRG-----------------------NQDGGDYRPRGNGYYRGGGR---------
AtG3BP-3  417 GGGRGGYGRTDFNGYGNNRGNNRGGYANRANGDGGGFPRANGNNGRVRRGGGNDANRATK
AtG3BP-4  395 ADDSK--------------------------TNGSAEDGEKEFKQVKSRRN----RKKSE
AtG3BP-5  389 SNGGRGYG-----------------------RNGSERRGESRNGEAHNGDGKVHQNGTVE
AtG3BP-6  390 VNGGRGYG-----------------------RRNSESNGD---GKAYQNNG----HGNTE
AtG3BP-7  425 G-----YG------YDYRRG---------GRGPGGGRSFN-------RRG-----NEYVA
HsG3BP-1  439 GGGMRG-----------------------------PPRGGMVQKPGFGVGRGLAPRQ---
HsG3BP-2a 442 GGGMMRDR----------------------DGRGPPPRGGMAQKLGSGRGTGQMEGRFTG
HsG3BP-2b 409 GGGMMRDR----------------------DGRGPPPRGGMAQKLGSGRGTGQMEGRFTG
consensus 481 ...  ...                         . . ..   .  .  .. .      .

AtG3BP-1  456 GGGSS-------
AtG3BP-2      ------------
AtG3BP-3  477 PVDDAPRVSVAA
AtG3BP-4  425 AAH---------
AtG3BP-5  426 ASR---------
AtG3BP-6  420 AKN---------
AtG3BP-7  454 SINSY-------
HsG3BP-1      ------------
HsG3BP-2a 480 QRR---------
HsG3BP-2b 447 QRR---------
consensus 541
```

Supplementary Figure S1 Clustal W alignment of the full-length amino acid sequences of all AtG3BPs as well as the human G3BPs including a consensus sequence. Conserved residues are highlighted.

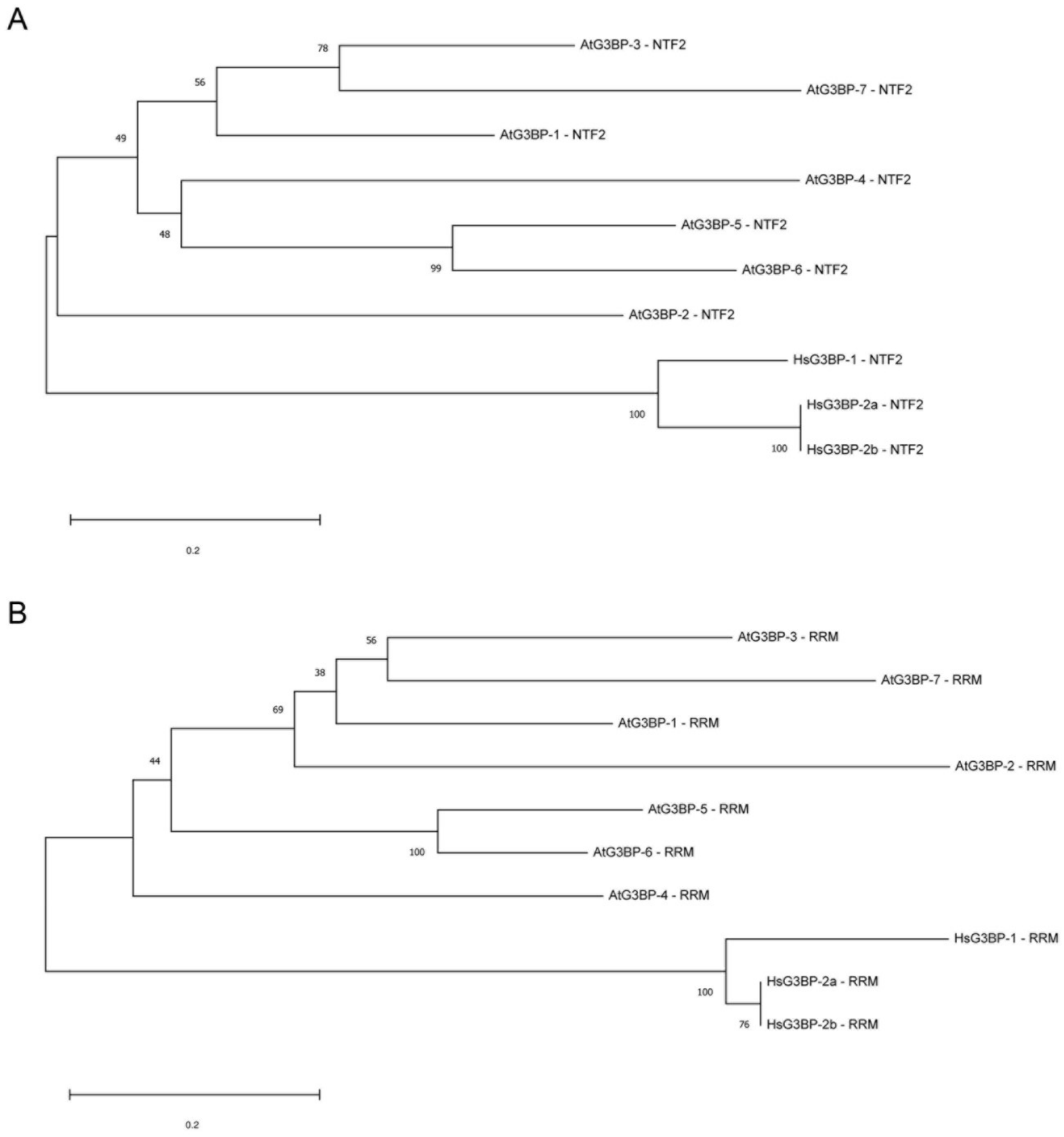

Supplementary Figure S2 Phylogenetic analysis of the full-length protein as well as the different domains of the human and Arabidopsis G3BP family.

The optimal tree with the sum of branch length of (A) 3.33018155 for the NTF2-like domain and (B) 3.31032469 for the RRM is shown. Evolutionary analyses were carried out as described in the methods section.

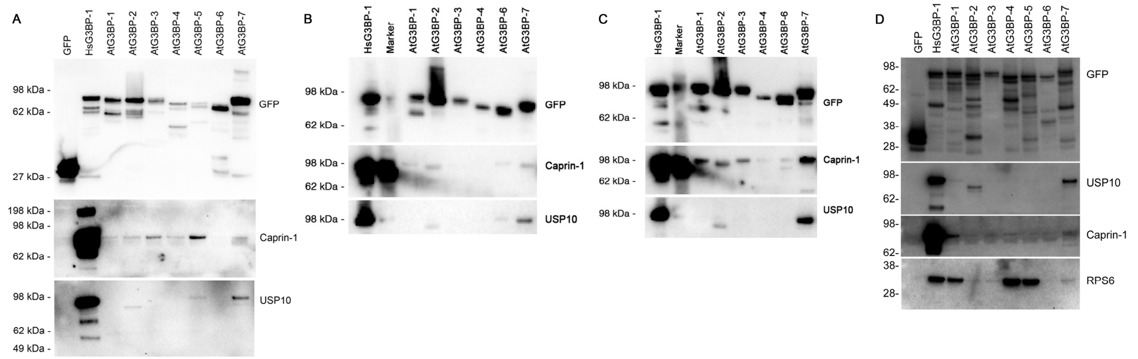

Supplementary Figure S3 Co-immunoprecipitation of human interaction partners in stable transformed (A-C) and transiently expressing (D) cell lines.

Anti-GFP co-immunoprecipitation for HsG3BP1/2$^{-/-}$ U2OS cells transfected with the different EGFP-G3BPs or free GFP. The western blot analysis of the indicated interaction partners has been carried out in four independent experiments. Please note that (B) and (C) lack the analysis for free GFP and AtG3BP-5 as these stable cell lines have not been generated at that point.

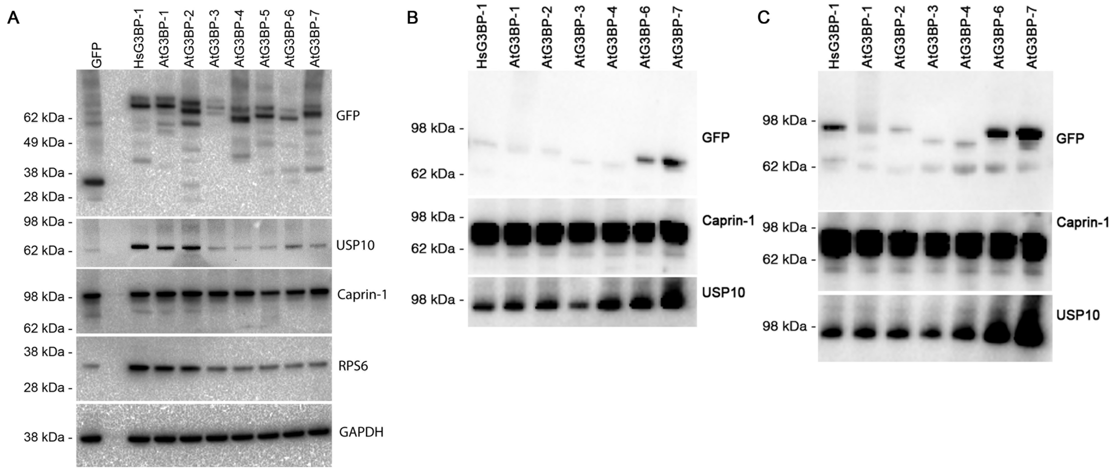

Supplementary Figure S4 Cell lysates referring to the co-immunoprecipitation of human interaction partners in cell lines expressing the indicated construct.
(A) Cell lysates referring to Figure S3D, (B) to Figure S3B, and (C) to Figure S3C. Please note that (B) and (C) lack the analysis for free GFP and AtG3BP-5 as these stable cell lines have not been generated at that point.
